# Unusual localization of pilocytic astrocytoma at the foramen of Monro mimicking a colloid cyst: a case report and literature review

**DOI:** 10.3389/fonc.2025.1717175

**Published:** 2025-12-11

**Authors:** Arturs Balodis, Sigita Zalite, Inese Briede, Jurijs Nazarovs, Liga Jaunozolina, Evija Bergfelde, Tõnu Rätsep, Kaspars Auslands

**Affiliations:** 1 Institute of Diagnostic Radiology, Pauls Stradins Clinical University Hospital, Riga, Latvia; 2 Department of Radiology, Riga Stradins University, Riga, Latvia; 3 Faculty of Medicine and Life Sciences, University of Latvia, Riga, Latvia; 4 Faculty of Medicine, Riga Stradins University, Riga, Latvia; 5 Department of Pathology, Riga Stradins University, Riga, Latvia; 6 Center of Radiology, Riga East University Hospital, Riga, Latvia; 7 Clinic of Neurosurgery, Riga East University Hospital, Riga, Latvia; 8 Department of Neurosurgery, Tartu University Hospital, Tartu, Estonia; 9 Department of Neurology and Neurosurgery, Riga Stradins University, Riga, Latvia

**Keywords:** pilocytic astrocytoma, colloid cyst, foramen of Monro, intraventricular tumor, third ventricle, mimic, differential diagnosis

## Abstract

Pilocytic astrocytoma is a circumscribed central nervous system (CNS) WHO grade 1 glioma that typically arises in the cerebellum or optic–hypothalamic pathways; intraventricular occurrence at or near the foramen of Monro is exceptional and can mimic a colloid cyst. Although tissue diagnosis is ultimately required, certain imaging features should keep neoplasm in the differential when a “colloid cyst” is suspected. We report a 22-year-old man with a well-circumscribed, non-enhancing mass at the foramen of Monro initially favored radiologically as a colloid cyst; radiology–pathology correlation after microsurgical resection established pilocytic astrocytoma with characteristic histology and a supportive immunophenotype. This case emphasizes that location and CT density are not pathognomonic and that surgical planning for equivocal lesions at the foramen of Monro should preserve a route for tissue confirmation to secure the correct diagnosis and guide management.

## 1 Introduction

Lesions at the foramen of Monro are uncommon and most frequently represent colloid cysts—benign, slow-growing epithelial cysts that can precipitate acute obstructive hydrocephalus if untreated. Their CT appearance is often characteristic (rounded, well circumscribed, typically hyperdense), although MR signal is variable, and several intraventricular tumors—including central neurocytoma, subependymoma, ependymoma, and low-grade astrocytomas—may arise in this region and mimic a colloid cyst radiologically. Pilocytic astrocytoma (PA), a central nervous system (CNS) WHO grade 1 circumscribed glioma that typically occurs in the cerebellum and optic–hypothalamic pathways, is exceptionally rare within the lateral or third ventricles; when present at the foramen of Monro, its similarity to a colloid cyst can generate diagnostic uncertainty with direct implications for surgical planning. We describe a young adult with a PA at the foramen of Monro initially interpreted as a colloid cyst, and we emphasize the imaging pitfalls, the decisive radiology–pathology correlation, and the operative considerations, while contextualizing the case with a brief synthesis of the limited literature on intraventricular PA at this location.

## 2 Case description

**Figure 1:**
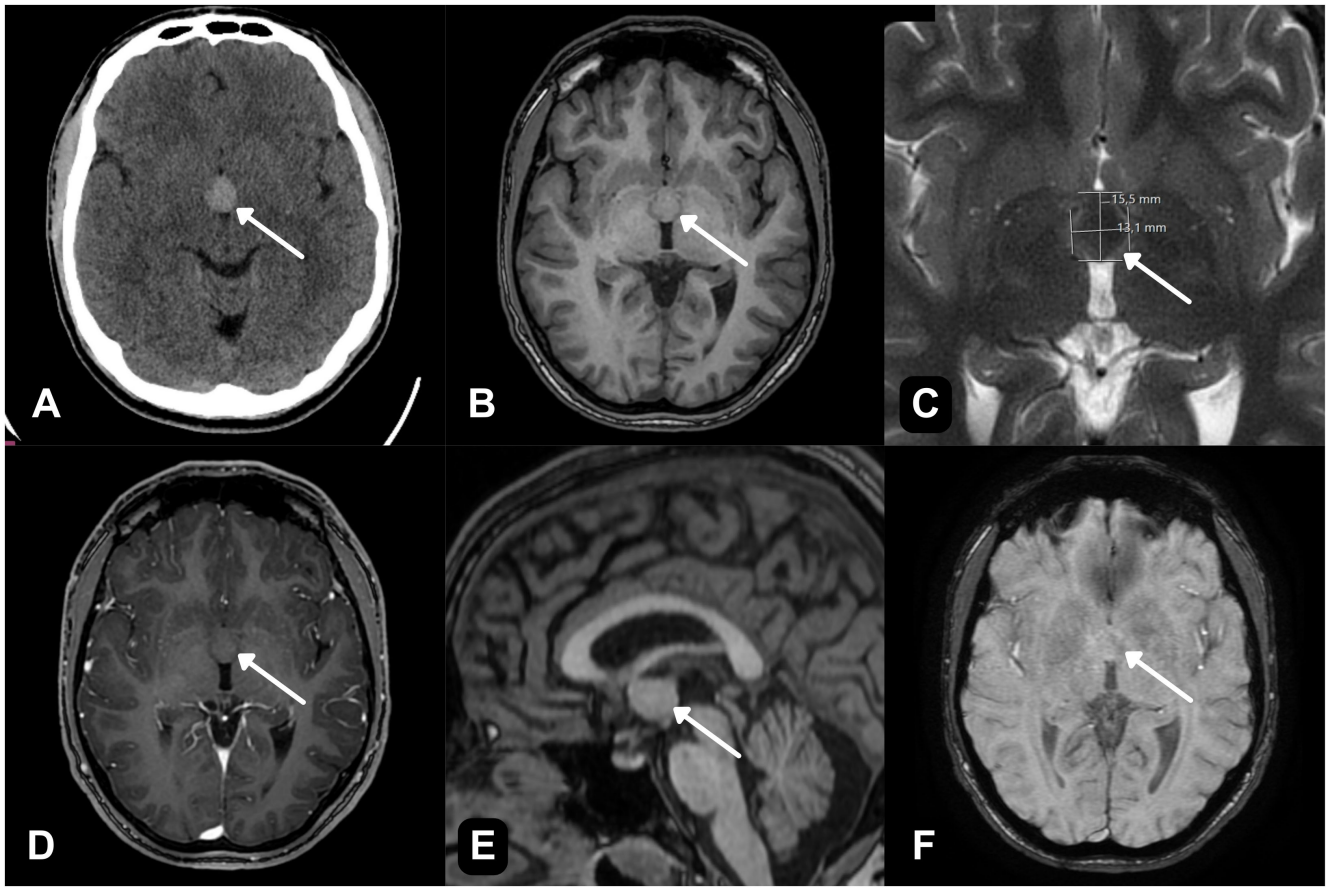
Initial CT and 1.5-T MR imaging. **(A)** Preoperative non-contrast CT demonstrates a hyperdense, well-defined intraventricular mass at the foramen of Monro (arrow). **(B)** Axial T1-weighted image shows a well-circumscribed lesion isointense to adjacent brain parenchyma within the anterior third ventricle (arrow). **(C)** Axial T2-weighted fast spin echo (FSE) image demonstrates a centrally hypointense area with a relatively hyperintense rim (arrow). **(D)** Axial post-contrast T1-weighted image shows no measurable enhancement (arrow). **(E)** Sagittal T1-weighted image confirms the lesion’s intraventricular location at the foramen of Monro (arrow). **(F)** SWAN/SWI shows no susceptibility to suggest intralesional hemorrhage or calcification (arrow).

A 22-year-old man was admitted after a head trauma. Non-contrast head CT performed at admission demonstrated a well-circumscribed, hyperdense intraventricular mass in the anterior third ventricle at the level of the foramen of Monro, measuring anteroposterior (AP) 15.5 × laterolateral (left–right) dimension (LL) 13.1 mm (**Figure 1A**). The Evans index was 0.25 with no ventriculomegaly or other CT signs of hydrocephalus, and there were no acute traumatic findings. The CT appearance (a rounded, well-defined, hyperdense lesion in the anterior third ventricle at the level of the foramen of Monro) was considered most compatible with a large colloid cyst. In the subsequent clinical course, the patient reported recurrent headaches and intermittent episodes of impaired coordination. Neurological examination at admission did not demonstrate gross focal deficits. Given the incidental finding, further evaluation with a brain MRI was undertaken.

**Figure 2:**
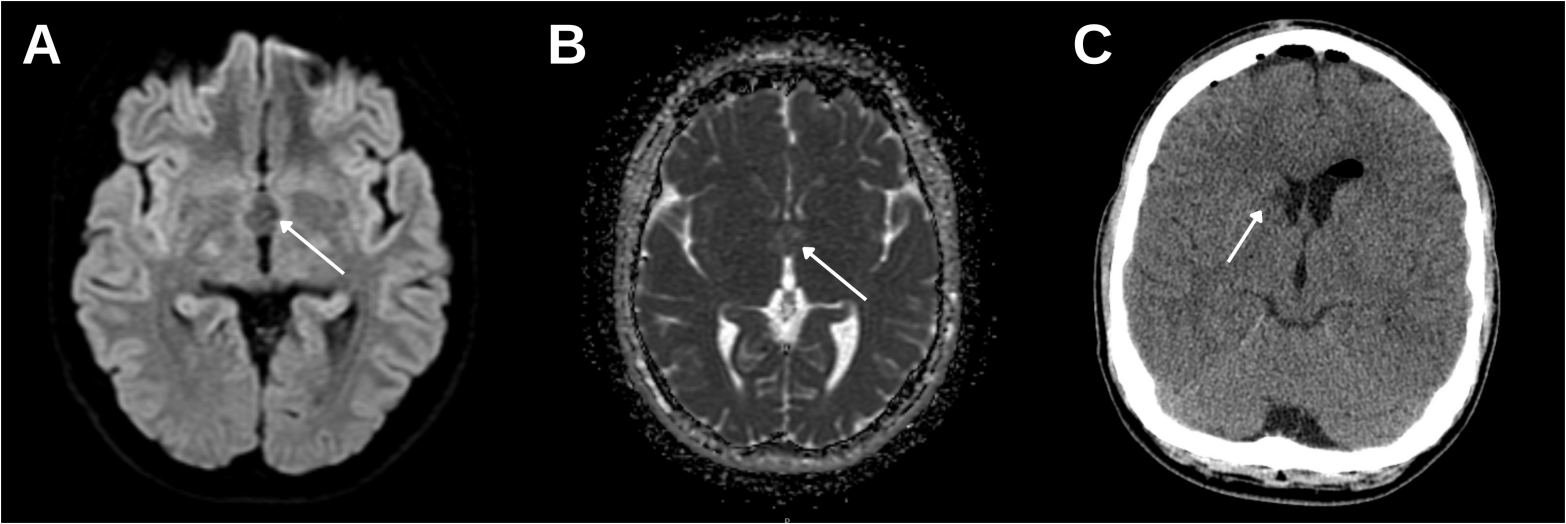
MR diffusion imaging using 1.5 T and postoperative CT. **(A)** Axial DWI shows no evidence of restricted diffusion. **(B)** Corresponding ADC map shows no reduction in ADC value; no imaging features suspicious for malignant transformation are seen. **(C)** Postoperative non-contrast head CT shows interval removal of the intraventricular mass with expected changes around the foramen of Monro; minimal postoperative edema is present at the caudate head (arrow), with no acute hemorrhage or ventriculomegaly.

Brain MR imaging was performed on a 1.5-T system, and it demonstrated a sharply marginated, smooth, cystic lesion measuring AP 1.5 × LL 1.4 × craniocaudal (CC) 1.3 cm. On T1-weighted images, the lesion was isointense to adjacent parenchyma (**Figure 1B**), and sagittal T1 confirmed its intraventricular location at the foramen of Monro (**Figure 1E**). On T2/fluid-attenuated inversion recovery (FLAIR), there was a centrally hypointense area with a relatively hyperintense rim (**Figure 1C**). Post-contrast T1 showed no measurable enhancement (**Figure 1D**). susceptibility-weighted angiography / susceptibility-weighted imaging (SWAN/SWI) revealed no susceptibility to suggest hemorrhage or calcification (**Figure 1F**). diffusion-weighted imaging (DWI) showed no restricted diffusion, and the apparent diffusion coefficient (ADC) map demonstrated no low values (**Figures 2A, B**). The lateral ventricles were asymmetric (left slightly larger) without periventricular edema or other signs of hydrocephalus.

Given the characteristic location and CT hyperdensity (**Figure 1A**), a colloid cyst was initially favored radiologically; however, the solid, cystic-appearing lesion without enhancement, together with its intermediate T2/FLAIR signal profile, kept an intraventricular neoplasm within the preoperative differential.

Under general anesthesia with neuronavigation, a right frontal osteoplastic craniotomy was performed. After dural opening, a microsurgical transcortical, transventricular approach was used to reach the foramen of Monro. A well-circumscribed intraventricular mass centered at the anterior third ventricle was identified. The lesion was resected microsurgically under high magnification with careful preservation of surrounding ventricular structures. Hemostasis was achieved, duraplasty was performed, and the bone flap was replaced.

The postoperative period was uneventful. The patient remained neurologically stable, with no new focal deficits. The surgical wound healed primarily, without signs of infection or cerebrospinal fluid leakage. He was discharged in good general condition with appropriate postoperative recommendations.

**Figure 3:**
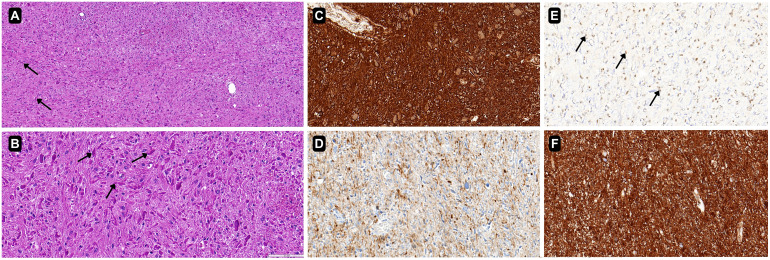
Hematoxylin–eosin (H&E)-stained sections show a biphasic glioma with compact piloid areas rich in Rosenthal fibers (block arrows) alternating with looser microcystic regions of spindle to bipolar astrocytic cells. **(A)** H&E, original magnification ×20. **(B)** H&E, original magnification ×40. **(C)** Diffuse GFAP positivity. **(D)** ATRX nuclear expression retained (arrows). **(E)** Strong nuclear SOX10 labeling. **(F)** S100 dense positivity. Panels C–F, original magnification ×40.

Hematoxylin–eosin-stained slides showed a moderately cellular, biphasic glioma with compact piloid areas containing abundant Rosenthal fibers and looser microcystic areas composed of spindle to bipolar astrocytic cells (**Figures 3A, B**). These features are characteristic of pilocytic astrocytoma (CNS WHO grade 1).

Immunohistochemical staining demonstrated diffuse glial fibrillary acidic protein (GFAP) positivity and S100 expression, with strong SOX10 nuclear labeling in tumor cells (**Figures 3C, E, F**). SOX10 positivity supports PA diagnosis and helps distinguish it from ependymoma. Nuclear ATRX expression was retained, and IDH1 R132H immunostaining was negative, suggesting diagnosis of a pilocytic astrocytoma and arguing against an isocitrate dehydrogenase (IDH)-mutant diffuse astrocytoma. Lack of CD31 and CD34 expression within tumor cells, but positivity within inner blood vessel endothelium, is consistent with a diagnosis of non-glioneuronal, non-vascular neoplasm; CD34 negativity in tumor cells is compatible with PA. The Ki-67 labeling index was <2%, in keeping with a low-grade lesion.

The histological assessment was independently reviewed twice by two experienced pathologists, both confirming the diagnosis.

## 3 Discussion

Colloid cysts are benign epithelial-lined lesions that account for less than 2% of all primary brain tumors, and more than 99% arise at the rostral third ventricle at or near the foramen of Monro (1). Most patients are diagnosed between the third and seventh decades of life, although cases have been reported in infancy. Classically, these cysts appear as a well-delineated hyperdense mass on non-contrast CT. MR signal is notoriously variable—approximately half are T1-hyperintense, and T2 signal ranges from hypo- to iso-/hyperintense; peripheral rim enhancement may be present but is not universal (2). This variability reflects cyst contents (protein/cholesterol/viscosity) and limits the specificity of MR alone (2, 3).

PA is classified as a grade I astrocytoma in the 2021 WHO classification of central nervous system tumors due to its benign biological behavior, limited growth, and slow progression (4). PA constitutes approximately 5% of CNS gliomas and predominantly affects children and adolescents aged 10–20 years, with no significant sex difference in incidence (5). The common onset site of PA is in the cerebellum and periventricular zone. Intraventricular location is uncommon.

In our patient, preoperative imaging favored a colloid cyst because of the location at the foramen of Monro and CT hyperdensity, yet the combination of T1 isointensity, intermediate T2/FLAIR with a centrally hypointense core, no measurable enhancement, no diffusion restriction, and no susceptibility on SWI did not exclude a neoplasm. The lesion proved to be a pilocytic astrocytoma (CNS WHO grade 1) on histopathology, illustrating how imaging overlap in this region can lead to misclassification and why radiology–pathology correlation is decisive when features straddle the cystic–neoplastic boundary.

This presentation is exceptionally rare. To our knowledge, the literature documents four cases across three publications in which PA at/near the foramen of Monro was radiologically favored as a colloid cyst: Missler et al. (2009) and Sharifi et al. (2018), together with a recent 2024 report describing a PA at the foramen of Monro first read as a colloid cyst. These cases are summarized in **Table 1**.

**Table 1:** Reported pilocytic astrocytomas at/near the foramen of Monro mimicking a colloid cyst.

Study	Age/sex	Hydrocephalus	NCCT	MRI T1	MRI T2/FLAIR	Enhancement
Missler et al. (2009) (28)	39/F	Yes	Isodense	Isointense	Hyperintense	None
Sharifi et al. (2018) (29)	21/F	Yes	Isodense with hypodense core	Hyperintense	Isointense with hypointense core	None
Sharifi et al. (2018) (29)	12/M	Yes	NR	Iso–hypointense	Iso–hypointense	Present
Algarni et al. (2024) (30)	42/F	No	NR	Isointense	Minimally hyperintense	None
Present case	22/M	No	Hyperdense	Isointense	Hypointense core with hyperintense rim	None

NR, not reported; NCCT, non-contrast CT.

Clinical presentation at the foramen of Monro is dominated by cerebrospinal fluid (CSF) flow dynamics and ranges from incidental findings to acute neurologic deterioration. Colloid cysts classically cause paroxysmal, often positional headaches (seconds–minutes), sometimes with nausea/vomiting, syncope/drop attacks, memory disturbance from fornical compression, and, in rare cases, sudden death due to abrupt obstructive hydrocephalus (6–8). In contrast, intraventricular pilocytic astrocytomas are typically symptomatic when hydrocephalus develops, most often with headache, nausea/vomiting, and gait/coordination difficulties; symptoms may be minimal or non-specific in the absence of ventriculomegaly (9, 10). In keeping with this spectrum, our patient was asymptomatic prior to head trauma, and the lesion was discovered incidentally on non-contrast CT. The absence of ventriculomegaly (Evans index 0.25) and a non-focal neurologic examination likely account for the lack of pressure-related or fornical symptoms. This underscores that clinical silence does not exclude neoplasm in the foramen of Monro region and supports maintaining a broad preoperative differential and a surgical plan that preserves tissue diagnosis when imaging is non-classic for colloid cyst.

Although PA is a CNS WHO grade 1 tumor, its behavior is not uniformly indolent. Outcomes are best after gross total resection (GTR): in a pediatric GTR series, 5-year event-free survival is reported at 95% and overall survival at 100% (11). In mixed-age surgical cohorts, 1/2/5-year progression-free survival is reported at 88%/80%/77% and overall survival at 93%/91%/87%, with recurrence approximately 11% after GTR versus ~45%–53% without GTR (12). Malignant transformation is rare, most often reported after radiotherapy, although sporadic cases without prior irradiation have also been described (13–15).

Histopathology in our patient showed a circumscribed, biphasic astrocytic neoplasm with abundant Rosenthal fibers and a low proliferative index (Ki-67 < 2%), establishing pilocytic astrocytoma (CNS WHO grade 1). The immunophenotype—diffuse GFAP and S100, strong nuclear SOX10, retained ATRX, and negative IDH1 R132H with tumor-cell negativity for CD31/CD34—supports a circumscribed astrocytic tumor and argues against IDH-mutant diffuse astrocytoma, ependymoma (typically SOX10-negative and often epithelial membrane antigen (EMA)-dot positive), and central neurocytoma/glioneuronal tumors (which would show neuronal marker expression) (16, 17). The lack of diffusion restriction and absence of measurable enhancement on MR are concordant with the tumor’s low cellularity/low proliferative activity and a relatively intact blood–brain barrier, while the centrally low T2/FLAIR signal plausibly corresponds to compact piloid, Rosenthal fiber-rich regions rather than simple fluid (18). This radiology–pathology alignment explains how a lesion at the foramen of Monro can mimic a colloid cyst preoperatively yet represent a low-grade neoplasm on tissue diagnosis.

Accurate preoperative classification matters for surgical planning. Whereas many colloid cysts are amenable to endoscopic techniques, intraventricular neoplasms (including PA) often warrant microsurgical resection to secure tissue diagnosis and achieve durable removal. Contemporary comparative analyses indicate that microsurgery attains higher gross total resection and lower recurrence/reoperation than endoscopy, albeit with potentially higher morbidity, while endoscopic outcomes continue to improve. Consequently, when imaging is equivocal, a strategy that preserves a safe route for tissue confirmation without compromising oncologic goals is prudent (19–21). In our patient, equivocal MR features and the need for tissue diagnosis led to a neuronavigation-guided right frontal microsurgical transventricular approach; postoperative CT showed interval removal of the intraventricular mass without acute hemorrhage or ventriculomegaly.

For residual, unresectable, or recurrent adult PA, active surveillance with serial imaging is reasonable when the disease is indolent and asymptomatic. Radiotherapy, either conformal RT or stereotactic radiosurgery (SRS), can provide durable local control/delay progression in selected residual or recurrent lesions, although late toxicities must be weighed (22–24). Systemic therapy in adults is not standardized: historical chemotherapy has shown modest activity (reported 5-year progression-free survival (PFS) roughly 34%–45% in low-grade glioma regimens), while modern strategies increasingly target MAPK pathway alterations that drive PA—MEK inhibition (selumetinib) and BRAFV600E-directed therapy (dabrafenib + trametinib) have demonstrated meaningful responses and improved PFS in prospective trials, supporting consideration of targeted therapy when actionable mutations are present (adult evidence remains limited and often extrapolated) (25–27).

### 3.1 Take-home message

A mass at the foramen of Monro that is hyperdense on CT and non-enhancing on MR is not pathognomonic for a colloid cyst; intraventricular pilocytic astrocytoma (CNS WHO grade 1) can mimic a colloid cyst, even without hydrocephalus. When MR shows T1 isointensity, intermediate T2/FLAIR with a centrally low-signal core, no diffusion restriction, and no SWI susceptibility, the appearance is non-classic for colloid cyst and should prompt consideration of neoplasm; in such inconclusive cases, select a surgical approach that preserves tissue diagnosis while allowing definitive resection if the lesion proves neoplastic.

## 4 Case summary

A well-circumscribed mass at the foramen of Monro is most often a colloid cyst, but imaging features overlap with intraventricular neoplasms and are not pathognomonic.

Colloid cysts are typically hyperdense on non-contrast CT; on MR, their T1/T2 signal is variable, so MR appearance alone cannot confidently exclude tumor, most notably PA, which can mimic this appearance.

PA is a CNS WHO grade 1 circumscribed glioma; while classically cerebellar or optic–hypothalamic, intraventricular PA is rare and may mimic a colloid cyst at the foramen of Monro.

When a “colloid cyst” is suspected, non-classic MR features—T1 isointensity/low signal, intermediate T2/FLAIR with a central low-signal core, absence of diffusion restriction, no susceptibility on SWI, and no measurable enhancement—should keep neoplasm in the differential.

Definitive diagnosis requires histopathology. PA is characterized by biphasic architecture with Rosenthal fibers and a supportive immunophenotype (GFAP/SOX10/S100 positive; ATRX retained; IDH1 R132H negative; low Ki-67).

Endoscopic strategies often suit colloid cysts, whereas suspected neoplasm typically warrants a microsurgical corridor that enables tissue diagnosis and the potential for gross total resection.

## Data Availability

The raw data supporting the conclusions of this article will be made available by the authors, without undue reservation.
